# Professional identity formation of female doctors in Japan – gap between the married and unmarried

**DOI:** 10.1186/s12909-019-1479-0

**Published:** 2019-02-12

**Authors:** Tomoko Matsui, Motoki Sato, Yoko Kato, Hiroshi Nishigori

**Affiliations:** 1 Department of General Medicine/Family and Community Medicine, Nagoya University Graduate School of Medicine, 65-banchi, Tsurumai-cho, Showa-ku, Nagoya, Aichi 466-8550 Japan; 2 Department of General Medicine Nagoya University, Nagoya University Hospital, Nagoya, Japan; 3 Department of Psychology, Sugiyama Jogakuen University, City, Nisshin, Japan; 4 Medical Education Center, Kyoto University Graduate School of Medicine, Kyoto, Japan

**Keywords:** Professional identity formation, Female physician, Gender roles, Gender stereotype, Masculinity, Personal identity formation

## Abstract

**Background:**

During professional identity formation (PIF), medical students and young doctors enter the process of socialization in medicine with their preexisting personal identities. Here, the authors focused on how gender influences both the professional and personal identities of doctors. The authors’ particular research question was how the professional and personal identities of female doctors are formed in Japan, a patriarchal and highly masculinized country, especially before and after marriage and childbirth.

**Methods:**

Narrative inquiry was used as the research methodology. The authors purposively sampled 10 unmarried and 15 married Japanese female physicians with varying lengths of full-time work experience and conducted individual semi-structured face-to-face interviews between July 2013 and February 2015. The authors recorded, transcribed and anonymized the narrative data and extracted themes and representative narratives related to the formation of professional and personal identities. Based on these, the authors developed the master narrative for the whole study.

**Results:**

The PIF process by which female physicians integrate personal and professional identities was profoundly affected by gender stereotypes. Further, participant narratives revealed the existence of conflict between married and unmarried female doctors, which created a considerable gap between them.

**Conclusions:**

Female physicians lived with conflicting emotions in a chain of gender stereotype reinforcement. To overcome these issues, we propose that it is necessary to depart from a culture that determines merit based on a fixed sense of values, and instead develop a cultural system and work environment which allows the cultivation of a professional vision that accepts a wide variety of professional and personal identities, and a similarly wide variety of methods by which the two can be integrated.

## Background

Interest in professional identify formation in medical education has expanded to the point that it is now considered independent to its role as a component of medical professionalism [1, 2]. Professional identities represent the physician’s interpretation of what being a good doctor means and the manner in which he or she should behave [3]. Doctors’ professional identities are formed by the social role of doctor; that is, by social and cultural expectations of who physicians should be [4]. This process requires the integration of personal values, morals and other attributes, such as gender, race, personal characteristics, religion and culture. Individuals enter the process of socialization in medicine with their own personal identities, and their personal and professional identities are further developed thereafter [5].

Focusing on gender, researchers have investigated factors influencing female doctors’ difficulties in forming professional identities. In surgical academia, for instance, underrepresentation of female physicians has been considered due to a lack of role models and gender awareness as compared with male physicians [6]. A literature review studying female doctors’ choice or rejection of careers in academic medicine revealed a lack of adequate mentors and the experiencing of gender discrimination [7]. A qualitative study interviewing third-year female medical students described their adaptation process in a male-dominated medical culture [8]. However, little is known about the longitudinal process of female doctors’ professional and personal identity formation, especially in the postgraduate setting associated with marriage and childbirth.

We assumed that the male-dominated social structure of Japan [9] made it a suitable setting to explore this theme. Japanese culture is patriarchal, based on considerable awareness of a gender division in labor such that males should work and females should do household work; in particular, the role of motherhood is emphasized [10]. A survey showed that many Japanese women consider having children is the biggest merit of marriage, and most married couples expect children [11]. Accordingly, it is a commonly held notion in Japan that married couples have or will have children. In the medical context, although Japan has seen a rise in the number of female doctors [12], the overall percentage of female physicians (around 20%) remains lowest among all Organization for Economic Co-operation and Development (OECD) nations [13]. The current employment rate of female Japanese doctors drops to 76.0% by around age 36 [14], and only 30% of women return to full-time employment [15]. Frequently cited reasons why Japanese female doctors leave full-time positions are: 1) difficulties in balancing work with the responsibilities of childbirth and child rearing; 2) poor working conditions, including physical problems; 3) long working hours; and 4) inability to take paid holidays [16]. Japan’s highly masculinized status should therefore be useful to enrich discussion on cross-cultural issues into research on medical professionalism and professional identity formation [17].

Highlighting these issues, Tokyo Medical University, a large teaching institution, was recently found to have manipulated entrance examination scores to boost male student enrollment at the expense of female applicants [18]. There has been a clear gender discrimination against female doctors and medical students in medical education in Japan [19, 20].

Against these arguments and background, we focused here on the longitudinal process of professional and personal identity formation in female doctors in Japan, from undergraduate through postgraduate to continuous development. Our research question was how the professional and personal identities of female doctors are formed in Japan, a patriarchal and highly masculinized country, especially before and after marriage and childbirth.

## Methods

### Setting

The study was conducted in Japan, a characteristically patriarchal and highly masculinized country. As mentioned above, according to Hofstede’s analysis, Japan’s masculinity rating is 95%, the highest among OECD nations [9]. Among 311,205 doctors currently in practice, females account for 63,504 (20.4%) [12]. Despite recent increases, women accounted for only 32.5% in 2014 [21].

### Methodology

We adopted narrative inquiry as the research methodology. This research methodology, widely used in sociology and anthropology, has its origins in hermeneutics and phenomenology [22, 23]. Narrative inquiry entails the collection and analysis of data and the expression of research results in the form of stories [24]. It is based on a constructivist epistemology whose strength lies not in adopting a stance toward the elucidation of a sole truth but in the expression of certain aspects of reality in a narrative form.

### Participants and selection criteria

We recruited study participants using a purposive sampling method in which individuals, events, and scenes considered able to provide important information are specifically selected for [25]. We selected candidate participants through introductions by friends, acquaintances, and faculties via e-mail or telephone. We recruited female doctors who completed undergraduate medical education curricula in Japan. Selection criteria were 1) female doctors with experience of working as a doctor either full-time or part-time; and 2) 1 to 20 years out of medical school, for whom the lifestyle changes resulting from marriage and childbirth may be regarded as significant. We carefully selected candidates to avoid bias in terms of medical specialty. Furthermore we did not include candidates who appeared unwilling to talk about their professional career or personal matters. We did not ask about their sexual preferences. Of 27 potential candidates we contacted, 25 (10 unmarried and 15 married) female doctors agreed to participate in the present study.

### Data collection

We conducted a face-to-face semi-structured interview with each participant that lasted approximately one hour. The first author, TM, a married female doctor employed in the department of general medicine at Nagoya University Hospital who experienced marriage in August 2012 and childbirth in April 2015, conducted all interviews. We conducted interviews between July 2013 and May 2014 for unmarried participants and between March 2014 and February 2015 for married participants, continuing through the period of pregnancy of the interviewer. A complete list of questions asked during the interview is shown in Table 1.

**Table 1:** Questions asked of female Japanese doctors for semi-structured narrative interviews

Number	Question
1.	What does the idea of working mean to you?
2.	Has the feeling of satisfaction you derive from working changed since you became a doctor?
3.	Looking at the future, what kind of position or career would you like to build?
4.	What do you think about private life events?
5.	Are you currently experiencing any troubles between your work and private life? If so, please elaborate.
6.	Did the meaning of work change for you before and after marriage? If so, how?
7.	Did the meaning of work change for you before and after childbirth? If so, how?
8.	Do you sense a difference in work ethics among female doctors whose status (e.g., marital status) differs from your own? If so, at what sorts of times?
9.	What emotions do you experience or deal with when you sense such differences in work ethics?
10.	When confronted with the work ethic of someone whose status (e.g., marital status) differs from your own, do you sense a kinship between the person who has that ethic and your past or future self?

We recorded all interviews using a digital audio recorder, and all contents were transcribed by a third-party contractor and anonymized immediately after the interviews.

### Data analysis

First, the first author (TM) read through the data and extracted themes and representative narratives related to the formation of professional and personal identities. We conducted this process by analyzing individual experiences of conflicts that were conceivably involved in the two identity formation processes. We noted the emerged core concepts, and based on them, chose representative data from each interview. Second, together with TM, the second (MS), third (YK), and fourth (HN) authors examined the themes and representative narratives while reviewing the original data, and modified them where necessary. Third, TM created a tentative conceptual map that charted the relationships among the concepts and representative data. Fourth, MS, YK, and HN then re-examined these analyses and carefully checked whether the analyses were consistent with the original data. In cases where a new concept had emerged, TM included it into the analysis. Finally, all authors developed the master narrative based on the concepts and representative narratives for the whole study, and then translated them from Japanese to English.

## Results

### Participant characteristics

Marital status, years of clinical experience, specialty and place of employment of each participant are shown in Table 2. Eight of the 15 married participants had children, and an additional participant was pregnant. Below, we present our findings about the process of professional identity formation in female doctors in Japan and how it is engaged with personal identity formation.

**Table 2:** Participant characteristics of female Japanese doctors recruited for semi-structured narrative interviews

Doctor	Post-graduate Years	Marital status	Children	Specialty	Employment at time of interview
A	8	Unmarried	No	Internal medicine	University hospital
B	4	Unmarried	No	Surgery	University hospital
C	6	Unmarried^a^	No	Anesthesiology	University hospital
D	8	Unmarried	No	Psychiatry	Graduate student
E	8	Unmarried	No	Internal medicine	Graduate student
F	6	Unmarried^a^	No	Internal medicine	Clinic
G	6	Unmarried	No	Gynecology	University hospital
H	6	Unmarried	No	Ophthalmology	Hospital
I	13	Unmarried	No	Ophthalmology	Hospital
J	13	Unmarried	No	Dermatology	Hospital
K	7	Married	No	Internal medicine	Clinic
L	8	Married	Yes	Internal medicine	Child care leave
M	11	Married	No	Internal medicine	Hospital
N	8	Married	No	University teaching staff	University
O	8	Married	No	Psychiatry	Graduate student
P	17	Married	Yes	Gynecology	Clinic
Q	11	Married	Yes	Internal medicine	Hospital
R	9	Married	Yes	Pediatric medicine	Graduate Student
S	8	Married	No	Internal medicine	Graduate student
T	10	Married	Pregnant	Surgery	University hospital
U	12	Married	Yes	Anesthesiology	Child care leave
V	10	Married	Yes	Dermatology	University hospital
W	12	Married	No	Radiology	Hospital
X	3	Married	Yes	Junior Resident	University hospital
Y	16	Married	Yes	Ophthalmology	Clinic

^a^Unmarried but with a partner

All husbands of married female doctors were male, and no participants were divorced. Although we did not positively confirm whether the unmarried participants had partners, we surmised from the context of the interview that two did have partners, and listed them as such in Table 2.

#### Before marriage

The findings described below are based on the narratives of unmarried doctors and reflections of married doctors.

##### Becoming a “competent” doctor

After graduating from medical school, female residents prioritized being “competent” doctors above everything else in their postgraduate training, and unconsciously interrupted the formation of their own personal identities. Their motivations were, above all, to acquire the medical knowledge and skills necessary to become competent doctors. One participant said: “I didn’t give much thought to my own private affairs during that time (at the beginning of residency). I just thought of becoming a good physician so that I could practice internal medicine as an attending doctor.” (Doctor Q).

Another participant stated: “In real clinical practices, I’d been learning new things that I couldn’t learn from textbooks, and mastering them one by one made me so happy. I literally stayed overnight at the room for residents, sometimes for about a week, forgetting to eat and sleep.” (Doctor L).

##### Feelings about married female doctors who prioritize a domestic role

Unmarried female doctors felt stressed by the sudden increase in workload when married female doctors unexpectedly took time off to address childcare responsibilities, and feelings of aversion arose toward the married female doctors. These married female doctors who prioritized their responsibilities of housework and parenting can be perceived as “gender-stereotyped”, considering men as breadwinners and women as being responsible for domestic matters*.*

One participant mentioned: “(On accepting work when a married female doctor suddenly took time off) Actually, even though I was the one who struggled with having to cover [for her] … there is no such system that lessens my workload, and I just ended up feeling that I got a lot of trouble. In that sense, even if I were to have a kid myself, I don’t think that I would want to be a bother like that.” (Doctor A).

Furthermore, some unmarried female doctors looked down upon women who prioritized domestic roles, while others perceived married female doctors who prioritized household roles but continued to work as doctors even while burdening their colleagues as “extravagant” (*zeitaku*). This was expressed in the following comment: “I met a married female doctor with children while I worked at the university hospital, and I didn’t like the way she was working. There was always an air of not being fully committed, and I could tell that people saw her in that way too. It’s very depressing to think about it, but that’s the way you have to work (if you have children), and I feel like I don’t want to go down that route. I want to work in the position I am now, single, without feeling inferior to anyone”. (Doctor N).

Another woman answered: “Having both (work and private life), from my own perspective I feel that’s a bit extravagant, somehow”. (Doctor A).

Marriage for female physicians was seen as something that impedes working as a doctor, which was also expressed in the following comment: “… (Among attending doctors) there was a view that since female doctors would quit working once they got married, teaching them was a waste of time.” (Doctor W).

##### Consciousness of reproductive age and own marital status

Female doctors came to be conscious of their reproductive age and upon feeling a certain sense of accomplishment in their professional training. For them, the formation of personal identity was equated with getting married and becoming a mother, which conformed to gender stereotypes. Therefore, unmarried female doctors eventually came to confront a personal sense of inadequacy. One participant explained: “When I got around to the postgraduate year five (PGY-5), I realized that I was about to finish my residency, and so I started thinking calmly about what I wanted to do with the rest of my life. When I thought about how the people around me had married and were having children, it was probably during my PGY-6 that I started thinking about my future … the idea of possibly continuing to devote myself solely to the job was unsettling (for me).” (Doctor E).

Another woman elaborated that: “(Being highly evaluated as a doctor) is wonderful and important, and I think it would probably be tough not to be in such a situation … , but, suddenly, I began to wonder whether continuing with this lifestyle was really going to be a happy life or not. And then, for me, imagining a life in which I never have a family and just continue with this lifestyle until I die, that’s really terrible.” (Doctor B).

Furthermore, even in medical practice, being encouraged to conform to getting married, especially by male doctors, amplified such feelings of inadequacy. This view is expressed in the following comment: “A male doctor told me, ‘You shouldn’t work so hard. I’ve seen many female doctors who have worked so hard but ultimately ended up not married, alone forever. I think that happiness for male doctors and happiness for female doctors are different things. For a man to work hard as a doctor and for a woman to work hard as a doctor is different, so I think it’s better for you not to work too hard’.” (Doctor N).

##### Meaning of getting married and having children

Female doctors became increasingly aware of their reproductive age, which served to deepen a sense of impatience about getting married. For them, getting married and having a family was perceived as an urgent and important task to be accomplished, as expressed by one doctor: “If I’d given up on the idea of having children, then I probably wouldn’t be in such a hurry; I might not be so anxious, might not feel that I have to get married.” (Doctor E).

Another participant answered: “The only thing that I had not obtained (in my life) was marriage.” (Doctor S).

##### Conflict between being a doctor and being a woman before marriage

Through becoming aware of their own personal identity formation, unmarried female doctors felt torn between prioritizing being a doctor and being a woman who would become a wife and mother. One doctor explained: “The more I worked, the more responsibilities I had, which I felt comfortable with (as a doctor), but, if I was stuck at the hospital on Saturdays and Sundays … , then I don’t think I would be able to be truly happy. … (so) I held myself back a little bit (from my work), but I also felt guilty about it. Dating? Should I be dating? Those kinds of strange conflicts came up. I felt like I had two value systems, and there was a rift or division between them.” (Doctor N).

Another woman stated: “(In terms of marriage, I seriously considered) if I could just become like a man and find a stay-at-home husband. If I did that, the situation would be really the same (as the present style which prioritizes being a doctor), right? But truthfully, I wouldn’t like that. So, I could not be like a man ….” (Doctor E).

Moreover, female doctors found limitations in the way of life that prioritizes being a doctor (they had pursued thus far), and, considering their reproductive age, began seeking marriage partners or changing their professional careers. This is expressed in the following statements: “At a certain point, I felt I had to do something or I would never have chances (for having and raising children). That’s why I began looking for a spouse.” (Doctor T); “I thought I had to change my specialty because I thought it would be basically impossible to reconcile working at a hospital with having a family (if I remained in the present specialty).” (Doctor M).

#### After marriage and childbirth

The findings described below are based on the narratives by married female doctors.

##### Change in social approval and self-recognition following marriage

Some female doctors felt they had attained a certain degree of social approval through their marriages, as exemplified by the following statement: “Society really favored me once I got married, or rather I feel I gained a certain kind of recognition. I don’t mean recognition at work (as a doctor) either.” (Doctor N).

Moreover, they acquired a sense of relief from being able to develop their future careers as doctors. Or, as one participant put it: “I had been thinking for a long time that my career as a doctor would mainly depend on it (marriage), so I was really happy because I finally had my foundation.” (Doctor N).

##### Conflict between being a doctor and being a woman after marriage

After childbirth, the married female doctors came increasingly closer to prioritizing domestic matters, as expressed by one woman: “I had a strong feeling that I would not be satisfied if I did not take care of it (household affairs) by myself.” (Doctor R).

They dealt with a variety of emotions as a consequence, one of which was a sense of stagnation with regard to ‘stepping up’ as a doctor. As one participant answered: “I couldn’t see as many patients as I’d hoped to, or spend as much time as I wanted to in study, so I felt like I’d had my wings clipped (as a doctor) since I had my kid.” (Doctor U).

Moreover, as they worked less hours than other doctors, they felt they were in a minority within the doctor community, where members prioritized being a doctor over everything else. One participant commented: “There aren’t that many doctors (in the hospital I work at), so the number of night shifts (each doctor has to do) tends to increase when there are lots of these kinds of female doctors. So, I think there’s a kind of criticism from others. There’s an underlying assumption that you are a sufficient contributor (to the workplace) only if you can work night shifts.” (Doctor R).

Some female doctors felt guilty because their work styles that prioritized being mothers and limited their working time created a sense of inequality of workload among their colleagues. Others experienced a sense of shame, as though conforming to prioritizing being mothers and domestic matters was somehow an excuse that had released them from discharging their responsibilities as doctors. This is expressed in the following statement: “I guess, because of a shortage of doctors, I think I have to go back to working full-time (from part-time) as early as possible … I suppose I’m probably inconveniencing my colleagues.” (Doctor Y).

Another participant put it this way: “(Because I got pregnant), I was exempt from night shifts. … I didn’t really want to do it anyway, honestly. … On-Call was also quite stressful, so I didn’t like it. … And I can’t help but feel like I sort of ran away from it ….” (Doctor Q).

##### Influence of personal experience on being a doctor

Even while feeling perplexed at being a doctor who prioritizes her domestic roles, some female doctors also felt liberated from the sense of obligation that they had felt in their unmarried lives of having to prioritize being a doctor. As explained by one participant: “After having children, in my job, I began to receive consideration of my work hours from my boss so that I could go home around 5 p.m. every day. I think that was a big factor in being able to interact with patients in a more relaxed manner. (Before this happened, I felt I was too busy to take good care of my patients).” (Doctor V).

Furthermore, new experiences in private life, like childbirth and parenting, lead to enhanced empathy toward and approval by patients, which contributed to professional identity formation. By accepting themselves as maintaining the priority of their domestic role, the female doctors acquired a certain sense of self-affirmation. This was exemplified by the following statements: “(From the experience of being a mother and taking my sick child to the doctor,) it really hit me once again that everyone comes (to the hospital) because they want their kid to get better, and that’s all.” (Doctor V); “When I was working with outpatients, I could give off an image of a young, stubborn female doctor … , but the impression changed when I could show my non-doctor side (to my patients) by wearing my wedding ring or telling that I had kids.” (Doctor Q).

##### Relationship of unmarried female doctors and married female doctors with prioritization of domestic roles

However, married female doctors were conscious of their distinction from unmarried female doctors. The influence of personal identities formed through being a wife or a mother on professional identities was not easy to convey to unmarried female doctors, as can be understood from the following statement: “Working with unmarried doctors was actually a bit harder because it was between two women. It isn’t easy to say that I have kids as a reason or excuse (for not working hard like other full-time doctors). It would be easy to say, ‘I’m sure you don’t understand what it’s like,’ because they don’t have kids, but to say that to someone who doesn’t have kids—it just wouldn’t really get across, I think. So, I try not to say it at all.” (Doctor Q).

This was because they were aware of the change they experienced before and after marriage and childbirth, from prioritizing being a doctor to conforming more to prioritizing being a mother and domestic matters, and they believed, based on their personal experience, that the effect of being both a wife and a mother on being a doctor is not something that can be understood without being in that position. This thought was expressed in the following statements: “Before I was married, I felt a sense of incredulity at the idea of someone taking time off (to attend a child’s school sporting event). But (now that I have a child myself), I would definitely want them to go and would fill in for them myself.” (Doctor Y); “I think we really can’t know unless we have been in that position ourselves.” (Doctor R).

## Discussion

We found that the process by which female doctors integrated personal and professional identities was profoundly affected by gender stereotypes that value marriage, childbirth and having a family and which prioritize domestic roles after marriage, namely being a wife and being a mother. We demonstrated that unmarried female doctors looked down upon married female doctors who established a personal identity which conformed to gender stereotypes. Even married female doctors used to have this feeling before they got married. Equally important, married female doctors did not actively talk to unmarried female doctors about the influence of being a wife and a mother on their professional identity. This was because they felt a sense of discrimination based on marital status. Such conflict and discrimination between married and unmarried female doctors created a gap between them. This in turn raises the question of why such a gap exists.

In our study, the married female doctors stated that the influence of the establishment of personal identity on professional identity was “impossible to understand if one has not experienced it.” They had developed a sense of guilt or puzzlement because they felt they were physicians who established a personal identity that conforms to gender stereotypes. We consider that the reason for this feeling lies in the deeply rooted value in medical culture that prioritizes professional identity over personal identity. Because this value seeps into and manifests itself inside a doctor’s mind, it is considered difficult for female doctors, especially married doctors, to attain adequate self-affirmation. This likely explains why married female doctors did not actively share their experiences with unmarried female physicians, who prioritized the establishment of professional identity over personal identity formation.

Another important factor that created a gap was the deeply rooted belief that women must form a personal identity which conforms to gender stereotypes that value marriage, childbirth and having a family. Unmarried female doctors had a sense of inadequacy as being a minority because they felt they were women who did not conform to gender stereotypes. We consider that the origin of this feeling lies in values shared in society - in this case, Japanese society. Previous studies have shown that female residents were more family-oriented than male residents [26] and that female physicians experienced discrimination based on gender in their workplace [27]. In our study, even in the participants’ workplaces, gender stereotypes were reinforced. Therefore, based on these values or gender stereotypes, it is considered to be difficult for female doctors, especially unmarried doctors, to attain adequate self-affirmation.

As a result, a gap between married and unmarried female physicians emerged. In other words, this gap developed because female doctors, both married and unmarried, felt that they were belonging to a minority based on two sets of values, one that prioritizes being a doctor in the medical field and another one that prioritizes conforming to gender stereotypes in Japanese society. Unmarried female doctors felt they were in a minority in terms of personal identity formation. Married female doctors felt that they in a minority in terms of professional identity formation. This gap was created because the two values exist in parallel dichotomy, which rejects diversity of both gender roles and work values of doctors.

In Japan, the existing job support system for female physicians is mainly targeted toward married female physicians who are raising children [28]. Such a support system may duplicate the misconception that family-related challenges in medicine, such as working long hours or taking time off to care for children, are exclusively women’s issues and portray an idealized image of married life with children to young female physicians. We believe we have to recognize that this sort of support system may reinforce the two existing values, especially gender stereotypes.

Recent studies show that women researchers and female academics are facing similar identity- and career-related challenges [29–33]. In addition, a study showed that childbearing and raising is perceived as a threat to career advancement in female residents in the United States [34]. Further, even in a country with a very low masculinity, Norwegian women doctors tend to choose or change their specialties in order to accommodate work and family life [35, 36]. Therefore we hope the current study will encourage the medical community, including both male and female physicians around the world, to engage in a dialogue about how the concepts of professional identity formation and gender stereotypes should be re-evaluated. We believe that we should develop a cultural system and work environment in which the polarity of being excellent at both doctoring and parenting can be managed.

We conducted this study in a single country and with a relatively small sample size, which might limit the transferability and generalizability of our findings. However, the small sample size enabled the use of comprehensive in-depth interviews. In addition, we did not ask about sexual preference when we recruited the participants. Therefore, we were not able to clarify how homosexual female doctors perceived their professional identity formation process. Furthermore, we did not compare married female physicians with and without children, although half of the married study participants did not have children. As stated above, Japan is a patriarchal society, so once a woman is married, she is faced with the major social expectation of a gender division in labor under which males should work and females should do household work, and in which wifehood and motherhood are often discussed together [37]. For this reason we did not discuss wifehood and motherhood separately, which is also another limitation of this study. Finally, in some instances, we might not have been able to elicit authentic responses from the interviewees because of their feelings toward the interviewer’s marital and maternal status at the time of the interview.

## Conclusions

It is deeply distressing that female physicians must continue to live with a chaos of conflicting values in the chain of gender stereotype reinforcement. In order to break this cycle, it is necessary to depart from a culture that determines merit based on a fixed sense of values, and instead develop a cultural system and work environment in which we can cultivate a professional vision that accepts a wide variety of professional and personal identities and just as many variations in how the two can be integrated. We believe that doing so will enhance professional identity formation that integrates personal identity based on various personalities and values, not solely on gender or marital status. Through this, we can create a medical community in which male and female professionals can work together with mutual respect and pride as physicians.

## Abbreviations

PIF: Professional identity formation
